# Mechanistic Modelling of Coupled UV Energy Penetration and Resin Flow Dynamics in Digital Light Processing (DLP)-Based Microfluidic Chip Printing

**DOI:** 10.3390/mi16020115

**Published:** 2025-01-21

**Authors:** Xinhui Wang, Antony Seng Kai Kho, Jinghang Liu, Tianyu Mao, Michael D. Gilchrist, Nan Zhang

**Affiliations:** 1 Centre of Micro/Nano Manufacturing Technology (MNMT-Dublin), School of Mechanical & Materials Engineering, University College Dublin, 4 Dublin, Ireland; xinhui.wang@ucdconnect.ie (X.W.); tianyu.mao@ucdconnect.ie (T.M.); michael.gilchrist@ucd.ie (M.D.G.); 2 School of Mechanical and Manufacturing Engineering, Dublin City University, 9 Dublin, Ireland; antony.kho@dcu.ie; 3 DCU Life Sciences Institute, Dublin City University, 9 Dublin, Ireland; 4 School of Mechanical Engineering, Technological University Dublin, Bolton Street, 1 Dublin, Ireland; jinghang.liu@tudublin.ie

**Keywords:** digital light processing, microfluidic chip, over-curing print, COMSOL Multiphysics 6.1

## Abstract

Digital light processing (DLP) technology has emerged as a promising approach for fabricating high-precision microfluidic chips due to its exceptional resolution and rapid prototyping capabilities. However, UV energy penetration and resin flow dynamics during layer-by-layer printing introduce significant challenges for microchannel printing, particularly in controlling microchannel over-curing. In this study, a novel 3D DLP over-curing interaction model (DLP-OCIM) was developed to investigate the coupled effects of UV energy penetration and directional resin flow on the over-cured structure formation of microchannels. COMSOL Multiphysics 6.1 simulations incorporating UV light propagation, photopolymerization kinetics, and resin flow dynamics revealed that microchannel over-curing is a result of both energy infiltration through previously cured layers and periodic resin flow induced by the peeling process. Experimental validation using linear and annular microfluidic chips demonstrated that increasing layer thickness induces progressive over-curing, leading to inclined cross-sectional structures. Additionally, the microchannel geometry and size significantly influence resin flow patterns, with shorter transverse microchannels producing flatter over-cured profiles compared to their longitudinal counterparts. This study provides the first comprehensive analysis of the dynamic interplay between UV energy penetration and resin flow during DLP-based microchannel fabrication, offering valuable process insights and optimization strategies for enhancing shape fidelity and printing accuracy in high-resolution microfluidic chips.

## 1. Introduction

Microfluidic chips are sophisticated platforms widely recognized as lab-on-a-chip systems, enabling the manipulation and analysis of fluids at the microscale through integrated structures such as microchannels, pumps, valves, reservoirs, and electrodes [1,2]. Since the early 1990s, microfluidic technologies have gained significant traction, driven by advancements in fabrication techniques that provide unparalleled efficiency in fluid control and processing [3]. Compared to conventional methods, microfluidic systems drastically reduce sample volume requirements, minimize reagent consumption, and shorten analysis times while simultaneously enhancing automation and reproducibility [4,5], which integrates principles and methodologies from biology [6,7], chemistry [8,9], medicine [10,11] and fluid mechanics [12,13]. In recent years, microfluidic chips have become indispensable in biological and medical research, supporting advanced analytical capabilities and enabling faster, more cost-effective, and accessible diagnostic solutions [14].

The fabrication techniques for microfluidic chips have evolved significantly in response to the growing demand for their diverse applications. Early fabrication methods primarily relied on polymers such as PMMA and ABS, using solvent-based techniques to create microchannels with high structural integrity [15]. However, these methods faced critical limitations, particularly in terms of optical transparency, which restricted their applicability in fields requiring optical analysis and imaging [16]. To address these challenges, the hot-pressing technique was developed, offering improved transparency and enhanced microchannel precision [17]. Nevertheless, the hot-pressing process involved multiple complex steps and suffered from inconsistent yields, rendering it unsuitable for large-scale production [18]. By the late 1990s, the introduction of polydimethylsiloxane (PDMS) and soft lithography revolutionized microfluidic chip fabrication. This breakthrough significantly simplified the production process while maintaining high precision in microchannel patterning [19]. However, despite its advantages, PDMS-based fabrication still requires a series of delicate and interconnected steps, including casting, plasma treatment, curing, demolding, and bonding to glass substrates. Any failure at a single stage can compromise the entire process, necessitating a complete restart. Consequently, while soft lithography remains a cornerstone of microfluidic chip production, its inherent complexity, prolonged processing time, and susceptibility to procedural errors continue to challenge large-scale industrial applications [20].

In recent years, digital light processing (DLP) has emerged as a promising additive manufacturing technology for fabricating complex microfluidic chips due to its exceptional printing precision, design flexibility, rapid fabrication capabilities, and mild processing conditions. As a next-generation fabrication method, DLP has gained widespread recognition as an efficient solution for producing intricate microfluidic structures [20,21,22]. Compared to traditional microfabrication techniques, DLP enables the direct printing of microchannels and entire microfluidic chips, allowing for more complex structural designs while simplifying the manufacturing process and reducing the number of fabrication steps [23,24]. DLP printing operates on the principle of ultraviolet (UV) light-induced photopolymerization, where projected UV light selectively cures a photosensitive resin layer by layer. However, controlling UV energy penetration during the printing process remains a critical challenge. As UV light passes through a printed layer, its energy is not fully absorbed and continues to penetrate into deeper regions. This becomes particularly problematic when printing the top-layer structures after completing the microchannel fabrication. Residual UV energy from previous layers can penetrate through the cured layers and accumulate within the microchannel regions. Once the accumulated UV energy surpasses the resin’s critical curing threshold, unintended polymerization occurs, leading to overcuring. This phenomenon compromises the dimensional accuracy of microchannels and adversely affects the overall quality of the printed microfluidic chips [25,26,27].

To accurately simulate UV energy penetration during the DLP printing process and achieve high-precision microfluidic chip fabrication, researchers have attempted to develop the mathematical models of photopolymerization. These models aim to calculate UV energy penetration variations between cured layers and elucidate the relationship between UV energy penetration and microchannel overcuring. By optimizing the composition and ratios of photopolymers, these models effectively reduce UV energy penetration during printing, control energy distribution, and minimize the formation of over-cured microchannel structures, thereby enhancing printing precision. In 2015, Gong et al. [28] proposed the “Photopolymer Resin Exposure Model” by integrating the Beer–Lambert law and Jacob’s working curve. This model quantitatively established the relationship between UV exposure energy, the number of printed layers, and the minimum printable microchannel height, enabling predictions of UV penetration depth and defining the minimum printable height for specific photopolymers. Building on this model, Gong et al. [29] successfully fabricated microchannels with cross-sectional dimensions of 18 µm × 20 µm in 2017 by incorporating appropriate UV absorbers and customizing photopolymer compositions. Similarly, in 2020, Van et al. [30] further applied the model to optimize the selection of UV absorbers and photoinitiators, thereby restricting UV penetration depth and improving photopolymer curing precision. This advancement facilitated the successful printing of microchannel arrays with enhanced structural accuracy. Li Yang, et al. [25]. extended the model by developing a differential analytical framework based on Jacob’s working curve, integrating UV absorbers into photocurable materials to fine-tune energy absorption. This refinement allowed for the derivation of theoretical DLP printing parameters, enabling the fabrication of intricate 3D microfluidic structures. In 2023, Luo Zhiming et al. introduced a manufacturing strategy based on the DZC-VPP process, refining the mathematical model to predict UV irradiance thresholds required for resin polymerization. By precisely adjusting critical printing parameters, including UV irradiance, exposure time, and projection area, they effectively controlled UV penetration into adjacent resin layers, successfully printing microchannels with cross-sectional dimensions as small as 20 µm × 20 µm [31].

**Figure 1:**
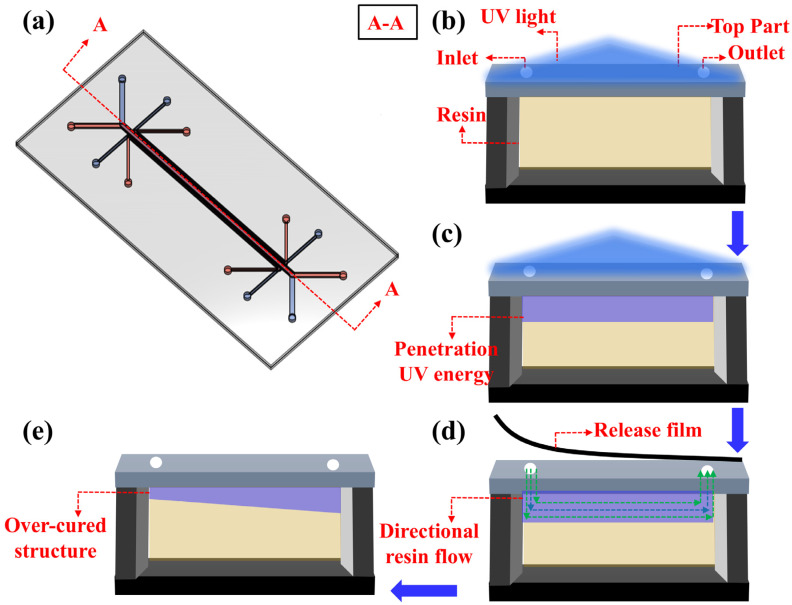
Mechanistic analysis of microchannel over-curing in microfluidic chips based on the DLP-OCIM model: (**a**) schematic representation of the overall structure of a traditional microfluidic chip, (**b**) cross-sectional view of a microchannel in the microfluidic chip, (**c**) UV energy penetration into the microchannel during the top layer printing process, (**d**) directional resin flow within the microchannel induced by the release film peeling process, (**e**) formation of over-cured structures in the microchannel resulting from the combined effects of resin flow and UV energy penetration.

Despite the significant progress achieved through the integration of the “Photopolymer Resin Exposure Model” in DLP-based microchannel printing, previous studies have predominantly focused on achieving microchannel heights below 30 µm while paying relatively little attention to the equally critical aspect of microchannel shape fidelity. In high-precision microfluidic chip fabrication, the accuracy of microchannel shapes is crucial, directly influencing chip functionality, such as mixing efficiency and fluid transport rates [32,33,34]. However, numerous studies characterizing high-resolution microchannel printing have revealed substantial deviations between printed microchannel geometries and intended designs due to overcuring effects, indicating that shape accuracy remains a significant challenge [27,29,35]. Furthermore, research on the formation mechanisms and key influencing factors of microchannel shape fidelity during the printing process has been relatively limited. The root cause of these challenges lies in two critical aspects. First, the traditional “Photopolymer Resin Exposure Model” is built on the assumption of unidirectional UV energy propagation, considering only vertical energy penetration while neglecting lateral energy diffusion and the three-dimensional energy distribution within microchannels. As a result, the model essentially constructs a two-dimensional planar energy penetration framework that fails to capture the complexity of light propagation behavior. Second, current DLP overcuring models almost entirely overlook essential dynamic factors inherent in the printing process, such as resin flow, resin redistribution, and the separation dynamics between the release film and the cured structure. These dynamic processes, which are unavoidable during actual printing, have a significant impact on energy accumulation and polymerization within microchannels. As shown in Figure 1, to address the limitations of existing models in explaining the over-curing mechanisms during the DLP-based microfluidic chip fabrication process, this study proposes a three-dimensional DLP over-curing interaction model (DLP-OCIM) based on COMSOL simulations. Figure 1a illustrates a traditional microfluidic chip, composed of interconnected hollow microchannel structures, while Figure 1b depicts the cross-sectional view of a microchannel. During the printing of the “Top Part” structure (Figure 1c), UV energy gradually penetrates into the resin within the microchannel. Furthermore, during each printing cycle, as shown in Figure 1d, the resin exposed to the penetrating energy undergoes directional flow, driven by the separation of the release film from the cured structure. With the layer-by-layer printing process, the continuous UV energy penetration and repeated resin directional flow ultimately result in the formation of an over-cured structure with a sloped profile within the microchannel (Figure 1e). The proposed COMSOL simulation model integrates the “General Form PDE”, “Domain ODEs and DAEs”, and “Particle Tracing for Fluid Flow” modules, enabling comprehensive simulations of UV energy penetration, energy distribution patterns, and resin flow directions within the microchannels. The COMSOL simulation model integrates the “General from PDE”, “Domain ODEs and DAEs”, and “Particle Tracing for Fluid Flow” modules, enabling a comprehensive simulation of UV energy penetration, energy distribution patterns, and directional resin flow within microchannels. This study systematically identifies over-cured structure formation as the result of coupled UV energy penetration and resin flow dynamics, offering a scientific basis for understanding the over-curing mechanism and its impact on shape fidelity. To validate the DLP-OCIM model’s accuracy, linear and annular microfluidic chips were designed, printed, and analyzed. Experimental results closely matched simulation predictions, confirming the model’s reliability and predictive power. This work represents the first systematic investigation into the interaction of energy propagation and resin dynamics within microchannels, providing theoretical insights and practical strategies for optimizing shape fidelity in high-resolution microchannel fabrication, addressing a critical gap in existing models.

## 2. Materials and Methods

### 2.1. Preparation of the Transparent Photopolymer

For microfluidic chip fabrication, high transparency of printing materials is a critical requirement, as microscopic observation of solution mixing and fluid dynamics within the channels is often necessary. However, the higher the transparency of the resin, the greater its UV energy-penetration capability, which significantly intensifies the over-curing phenomenon within microchannels. The transparent resin supplied by Phrozen Technologies Ltd. (Hsinchu City, Taiwan) was used to fabricate microfluidic chips. The transparency of the resin facilitates direct observation of the microchannel cross-sections in the printed microfluidic chips, enabling detailed evaluation of over-curing phenomena associated with increasing top-layer thickness. The resin primarily consists of acrylate oligomers, 4-(1-oxo-2-propenyl)morpholine, bis(1,2,2,6,6-pentamethyl-4-piperidyl)sebacate, and diphenyl(2,4,6-trimethylbenzoyl)phosphine oxide (<5%). Additionally, Brilliant Blue FCF dye was prepared by dissolving it in deionized water at a concentration of 0.1% (*w*/*v*) for microchannel visualization purposes.

### 2.2. Customized DLP Printing System

The customized DLP printing system features a custom digital light engine (Wintech Digital Systems Technology, San Marcos, CA, USA), providing a print area of 14.66 mm × 8.25 mm with a resolution of 5.4 µm. To enable high-precision printing, a custom *Z*-axis motorized stage drives the print platform vertically, achieving a bidirectional repeatability of less than 1 µm, allowing a minimum printable layer thickness of 10 µm and minimizing the stair-step effect in the cured structure. Additionally, a specially designed resin vat secures the release film (CS Hyde, Lake Villa, IL, USA), ensuring uniform contact with the resin surface and enhancing the flatness and precision of the cured structure. The resin vat assembly is mounted on a *Z*-axis manual translation stage (EJW11, YIHEDA Company, Dongguan, China) and an X/Y angular adjustment system (ELK56, YIHEDA Company, Dongguan, China). The X/Y angular adjustment system ensures precise alignment between the resin vat and the projection plane, while the *Z*-axis stage aligns the curing surface with the focal plane of the digital light engine, significantly improving printing accuracy and process controllability. This optimized hardware design not only improves the printing precision of microfluidic chips but also significantly reduces inherent system errors in DLP printing systems, enabling more direct observation of microchannel printing results and shape deviations caused by over-curing due to UV energy penetration.

### 2.3. Theoretical Model of Photopolymerization Based on DLP

As a foundational model in photopolymerization theory, Jacobs introduced the “Working Curve” model in 1992 to describe the variation in curing depth during linear laser scanning [36] (Equation (1)).

(1) $$C_{d}=D_{p}\ln\left(\frac{E_{max}}{E_{c}}\right)$$

In this model, the parameter $E_{max}$ defines the maximum energy absorbed by the resin during each layer’s exposure, which is the UV energy received at the resin surface. $C_{d}$ represents the curing depth of the resin layer while $D_{p}$ and $E_{c}$ correspond to the energy penetration depth and the energy threshold required for curing, respectively [36]. The penetration of light follows the Beer–Lambert law [37,38], where $D_{p}$ is determined by the absorbance characteristics and composition of the polymer system. It defines the depth at which the light intensity decreases to 1/e of its initial value due to absorption by the photopolymer resin. When the energy absorbed by the resin exceeds $E_{c}$, the resin initially transitions from a liquid state to a gel state, followed by further solidification, ultimately completing the curing process and achieving a full transformation from liquid to solid.

During the DLP printing process, as UV light traverses a print depth denoted as z, the attenuation of its luminous intensity $I_{\left(z\right)}$ is represented as follows:

(2) $$I_{\left(z\right)}=I_{o}e^{-\frac{z}{D_{P}}}$$

Then, the UV energy $E_{\left(z,t\right)}$ can be expressed as follows:

(3) $$E_{\left(z,t\right)}=I_{\left(z\right)}\cdot t=I_{o}te^{-\frac{z}{D_{P}}}$$

$z_{thickness}$ denotes the set printing thickness for each layer, t is the UV exposure time, while *n* represents the number of printed layers. By normalizing the printing thickness, the printing depth *z* is expressed as γ multiplied by the specified $z_{layer}$, written as $z=\gamma *z_{layer}$. Accordingly, the UV penetration energy during the printing of the *n*-th layer can be represented as follows:

(4) $$E_{n\left(\gamma ,t\right)}=I_{\left(z\right)}\cdot t=I_{0}te^{\frac{(\gamma -n)z_{thickness}}{D_{P}}}$$

The quotient of the gelation energy of $D_{n}$ for layer *n* is represented as follows:

(5) $$D_{n\left(\gamma ,t\right)}=\frac{I_{0}t}{E_{C}}e^{\left(\gamma -n\right)\frac{z_{thickness}}{D_{P}}}$$

By introducing λ and τ, the normalized energy dose $D_{n}$ can then be simplified.

(6) $$D_{n}\left(\gamma ,t\right)=\tau e^{\left(\gamma -n\right)\lambda }=e^{\left(\gamma -n+1\right)\lambda }$$

Equation (6) describes the variation of UV energy across consecutive layers, with (*γ* − *n* + 1) constrained within the range [0, 1]. During the DLP printing of microchannel top structures, UV energy does not dissipate immediately upon reaching the printed layer but instead gradually attenuates while penetrating deeper into the microchannel region, gradually causing over-curing. Although current technological methods can mitigate this effect to some extent, eliminating energy penetration entirely remains unattainable. This limitation arises because, in actual printing scenarios, UV exposure energy must exceed the minimum curing threshold required for the current printed layer to ensure strong adhesion between the newly cured layer and the previously cured structure. As a result, the energy supplied during printing often surpasses the theoretical requirement for curing a single layer, leading to inevitable over-curing. Thus, solely controlling UV exposure energy proves insufficient for completely preventing microchannel over-curing.

### 2.4. Determining and Optimization of Parameters for DLP Printing

To characterize the material properties of the transparent resin and determine optimal exposure time thresholds, the UV light intensity was fixed at 8.45 mW/cm^2^, and the cured layer thickness was measured at various exposure durations ranging from 1 to 5 s. After printing, the cured beam structures were examined using an optical microscope (Keyence, HX-5000, Osaka, Japan), with each layer’s thickness measured three times to ensure accuracy and reliability. The collected data were used to construct a “Jacobs Working Curve” model to describe the relationship between exposure time and cured thickness. A numerical interpolation method was applied to establish a precise correlation, enabling the accurate extraction of resin-specific parameters and optimal exposure time settings for the DLP printing process. This modelling approach improved prediction accuracy by determining the optimal exposure time and UV energy levels for the transparent resin, effectively reducing over-curing caused by excessive UV exposure while minimizing system errors from experimental variability and reducing the random measurement and printing deviations.

### 2.5. Closed Microfluidic Chip Design

**Figure 2:**
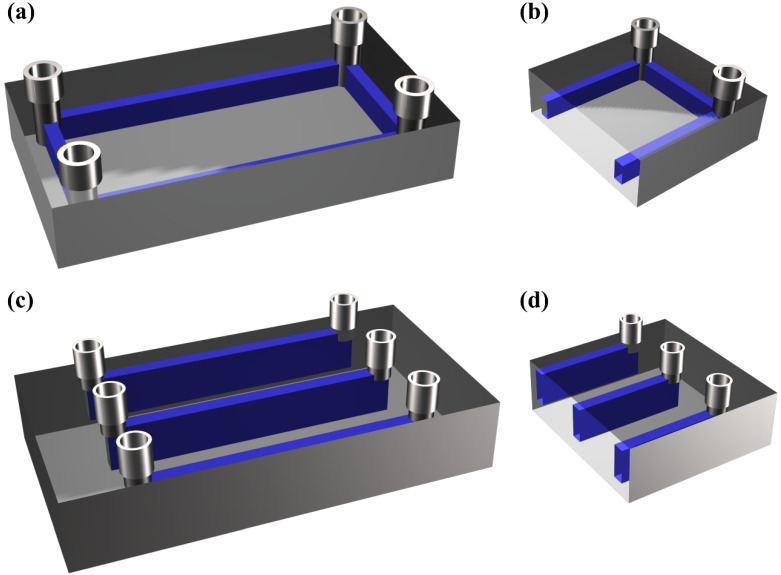
Microfluidic chip design: (**a**) the 3D model of the annular microfluidic chip, (**b**) the cross-section of annular microfluidic chip, (**c**) the 3D model of the linear microfluidic chip, (**d**) the cross-section of the linear microfluidic chip.

To investigate the effects of different microchannel structures and top-layer thicknesses on UV energy penetration-induced over-curing during the DLP printing process, two microfluidic chip designs were developed: an annular microfluidic chip structure (Figure 2a) and the linear microfluidic chip structure (Figure 2b). Both designs feature enclosed microchannels composed of three sections: the “substrate part”, the “microchannel part”, and the “top part”. The substrate height was uniformly set to 2.5 mm, while the microchannel height was fixed at 1 mm to prevent channel blockage and facilitate the observation of over-curing effects caused by varying top-layer thicknesses, which were adjusted from 0.1 mm to 0.5 mm to evaluate their impact on microchannel over-cured printing accuracy.

The linear microfluidic chip structure represents a conventional microfluidic chip design, featuring a narrow and elongated straight microchannel with an overall width of 0.5 mm and a length of 10 mm. This structure facilitates the analysis of the over-curing phenomena by examining the cross-sectional resin distribution within the microchannel under varying top-layer thicknesses, providing a means to assess the precision of printed geometries. However, this design of a linear microfluidic chip is inherently limited to investigating over-curing along a single axis, which does not fully capture the three-dimensional and dynamic nature of UV energy penetration during the DLP printing process, the limitations of which preclude a comprehensive and scientifically robust evaluation of over-curing mechanisms. To overcome this constraint and to study over-curing across multiple orientations, the annular microfluidic chip structure under identical printing parameters was designed and printed. The annular microfluidic chip features a characteristic annular microchannel configuration, which permits the systematic observation of over-curing distribution in all directions, as well as a more rigorous assessment of geometric accuracy in different orientations. Through comparative analysis of the over-cured structure of the linear and annular microfluidic chip structures, we want to develop a more comprehensive, systematic, and scientifically grounded understanding of the underlying mechanisms driving the over-curing phenomena during DLP-based microchannel fabrication.

### 2.6. COMSOL Simulation

To scientifically describe the dynamic mechanisms involved in DLP printing of microfluidic chips, particularly UV energy penetration, photopolymerization, and resin flow, this study developed a 3D simulation model using COMSOL Multiphysics 6.2. The DLP-OCIM integrates “General from PDE”, “Domain ODEs and DAEs”, and “Particle Tracing for Fluid Flow”, realistically simulating the whole over-curing process in enclosed microchannels during the printing process. UV light absorption through the microfluidic chip was described using the Beer–Lambert law, which is given by [39],

(7) $$\frac{\partial I}{\partial z}=\frac{I}{D_{p}}\;$$

where I is the UV light intensity, z represents the cured depth in the z-direction. The polymerization state was characterized based on the monomer conversion as a function of light intensity as described in the formulation proposed by [40]:

(8) $$\frac{\partial \left[M\right]}{\partial t}=-R_{p}=-\mathbb{P}\sqrt{I}\left[M\right]$$

where [M] denotes the monomer concentration of the photopolymer, t represents the UV exposure time, $R_{p}$ is the rate of polymerization, ℙ represents the overall rate constant for the photopolymerization process. Assuming the resin behaves as a laminar, incompressible, and Newtonian fluid, the fluid flow within the microchannel of the microfluidic chip was modeled using the continuity equation and the Navier–Stokes equations, expressed as the following:

(9) ρ∇·u=0

(10) $$\rho \frac{\partial u}{\partial t}+\rho \left(u\cdot \nabla u\right)=\nabla \cdot \left[\left(-p\right)I+\mu \left(\nabla u+{\left(\left(\nabla u\right)\right)}^{T}\right)\;\right]$$

Here, ρ represents the transparent resin’s density, u denotes the velocity field of the flow, p corresponds to the fluid pressure, μ refers to the dynamic viscosity, and I is the identity tensor. To comprehensively describe particle dynamics within the microchannel system, the motion of particles was modelled based on Newton’s second law, expressed as follows [41]:

(11) $$\frac{dq}{dt}=v$$

(12) $$\frac{d}{dt}\left(m_{p}v\right)=F_{t}$$

where q denotes the particle’s position, v its velocity, $m_{p}$ the particle’s mass, and $F_{t}$ the total force acting on it. Given the particles’ significantly small size and low relative velocity, the Stokes drag law ($F_{D}$) was applied, described as follows:

(13) $$F_{D}=\frac{1}{\tau_{p}}m_{p}\left(u-v\right)$$

Equation (8) incorporates key parameters such as the particle response time ($\tau_{p}$), the particle density ($\rho_{p}$), and the particle diameter ($d_{\rho }$), which collectively influence the drag force experienced by the particles.

(14) $$\tau_{p}=\frac{\rho_{p}d_{p}^{2}}{18\mu }$$

To model the UV photopolymerization and energy penetration during DLP printing of microfluidic chips, particularly for the top structures of microchannels, $I_{max}$ was defined as the incident light intensity at the top surface of each cured layer. Uniform initial values of I_0_ and monomer concentration ${\left[M\right]}_{0}$ were specified across all layers to ensure consistency in the simulation. For particle tracking, the inlet velocity u_0_ and particle entry conditions were prescribed at the inlet surface, while zero normal stress was applied as the boundary condition for all other open surfaces. The detailed definitions of all parameters used in the simulation are provided in Table 1.

## 3. Results and Discussion

### 3.1. Experimental Analysis Based on the Jacobs Working Curve

**Figure 3:**
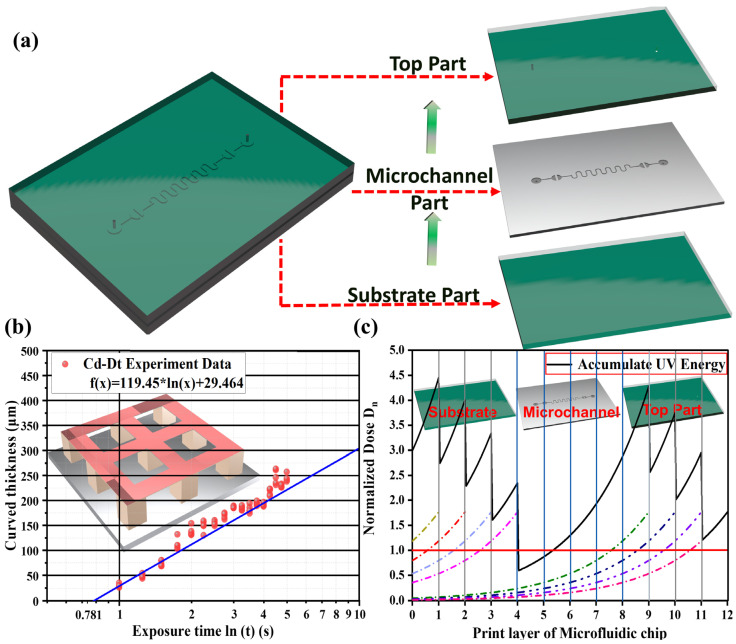
(**a**) Schematic of the microfluidic chip structure decomposition, (**b**) the experimental data of the exposure time with layer thickness, (**c**) accumulation and penetration of UV energy during microfluidic chip printing process-based DLP.

To ensure sufficient adhesion between consecutive layers, the actual exposure time was set to 1.2 times the theoretical value, resulting in a UV exposure time of 1.5 s. To accurately determine the required exposure energy for curing a 50 µm transparent resin layer, this study measured the cured thickness at different exposure times based on the “Working Curve” model, establishing the relationship between exposure time and cured thickness. Figure 3a shows the classical microfluidic chips, which typically consist of three main structural components: the “substrate part”, the “microchannel part”, and the “top part”. During the printing process, the substrate and microchannel sections are printed first, followed by the top structure. However, over-curing in microchannels primarily occurs during the printing of the top structure, where UV energy penetration poses a significant challenge. This unavoidable phenomenon complicates the fabrication of precise microchannels. Therefore, optimizing UV curing energy and fine-tuning printing parameters to minimize UV energy penetration are critical steps for achieving high-precision microfluidic chip fabrication. Firstly, the cured thickness was fitted as a function of exposure time. Therefore, to ensure measurement accuracy, a multi-mesh support column structure was designed (Figure 3b). This structure consists of multiple support and measurement units, each placed on a 0.5 mm thick substrate supported by two 2 mm high columns, ensuring stability and precision. Experimental results revealed that, for commercial transparent resin, the $D_{p}$ was 125.45, and the $T_{c}$ was 0.736. Calculations further determined that, under a UV intensity of 8.45 mW/cm^2^, the theoretical exposure time required to cure a 50 µm layer was 1.3 s. To ensure sufficient adhesion between consecutive layers, the actual exposure time was set to 1.2 times the theoretical value, resulting in a UV exposure time of 1.5 s.

With the transparent resin’s energy penetration depth and theoretical optimal UV curing time determined, these parameters were applied to the “Photopolymer Resin Exposure Model” for theoretical analysis. The analysis is illustrated in Figure 3c, showing that during DLP printing, layers 1 to 4 are cured first, corresponding to the “substrate part” of the chip. Since the microchannel structure is not yet printed at this stage, no over-curing occurs. After completing the substrate, the microchannel structure is printed (layers 5 to 8). During this phase, the microchannel region remains unexposed to UV light and thus does not absorb energy. However, once the microchannel structure is complete and the printing of the top part begins, UV energy penetration becomes the primary cause of over-curing. As shown in Figure 3c, during the printing of layers 9 to 12, residual UV energy continues to penetrate into the microchannel region after each layer is cured, gradually attenuating over time. Although the UV energy reaching the microchannel decreases as the layer count and cumulative thickness increase, the “Photopolymer Resin Exposure Model” indicates that, after printing the top four layers, the penetrated energy has already caused approximately 130 µm of over-curing. This means that for a microchannel designed with a height of 200 µm, the actual height is reduced to less than 70 µm. Such over-curing severely compromises the printing accuracy of microchannels and in extreme cases, can lead to channel blockages and eventual print failures. While the “Photopolymer Resin Exposure Model” effectively explains the relationship between energy penetration and over-curing thickness, it does not account for resin flow within the microchannel, a critical factor influencing shape fidelity. In fact, UV energy penetration is inherently a three-dimensional process. However, the “Photopolymer Resin Exposure Model” fails to represent the 3D dynamics of energy penetration, offering only a simplified mathematical description of energy attenuation and lacking the capability to depict the actual curing profiles within microchannels. To address these limitations, a comprehensive model that integrates energy penetration, photopolymerization reactions, and resin flow dynamics is essential. Such a model would provide a more complete and accurate representation of the over-curing formation process in microchannels and offer insights into the key factors influencing the development of over-cured structures.

### 3.2. Three-Dimensional Modelling of UV Energy Penetration in DLP 3D Printing

A comprehensive analysis of the three-dimensional dynamics of UV energy propagation is crucial for addressing the persistent challenges of over-curing during DLP-based microchannel fabrication. While the “Photopolymer Resin Exposure Model” serves as a foundational framework for interpreting resin curing induced by UV energy penetration, it provides only a simplified representation of the over-curing mechanism and its contributing factors. Specifically, this model primarily focuses on how a discrete point within the microchannel accumulates penetrated energy as successive top-layer structures are printed, leading to localized over-curing under ideal conditions. However, the spatial complexities and cumulative effects of UV energy propagation within the microchannel remain inadequately explored. These factors, including spatial irregularities and non-uniform energy distribution, play a critical role in shaping the resin curing profile. Understanding these dynamics is essential, as they significantly impact the dimensional accuracy, resolution, and functionality of the resulting microfluidic chips. Addressing these challenges requires a more detailed and robust model that accounts for the 3D propagation and interaction of UV energy within the microchannel, ultimately enabling the fabrication of high-resolution and precise microchannels.

**Figure 4:**
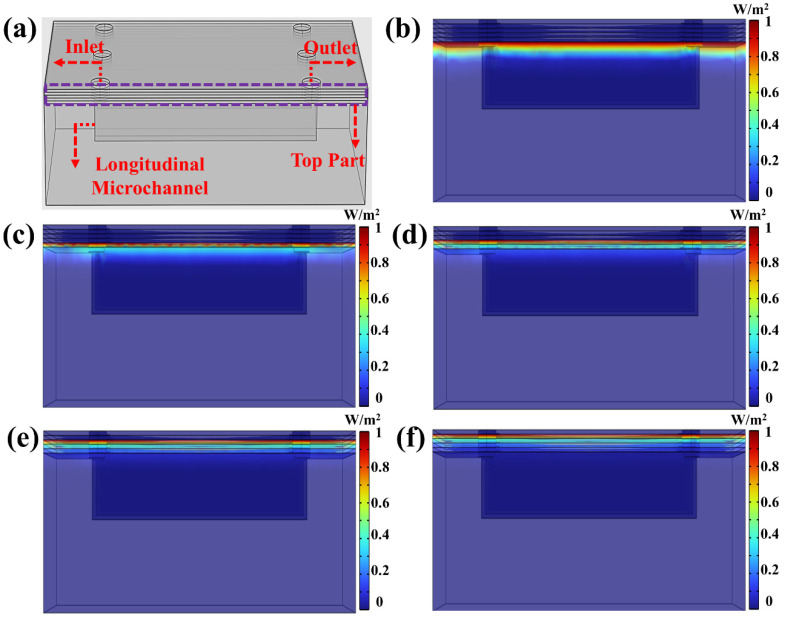
(**a**) Three-dimensional simulation of UV energy penetration in XZ cross-section of the linear microfluidic chip, (**b**–**d**) different printed top layer UV energy penetration: (**b**) top first layer, (**c**) top second layer, (**d**) top third layer, (**e**) top fourth layer, (**f**) top fifth layer.

Since both linear and annular microfluidic chips are fabricated under identical conditions—including the same DLP 3D printer, materials, layer thickness, and UV exposure energy—the UV energy penetration process is expected to be consistent across these structures. To simplify the analysis while maintaining a comprehensive understanding of energy penetration dynamics during the printing process, the linear microfluidic chip structure was selected as the observation model, as shown in Figure 4a. This selection allows for a clearer and more straightforward evaluation of the key factors influencing UV energy propagation and over-curing within the microchannels. The COMSOL’s “General Form PDE” module was employed to comprehensively simulate and evaluate the UV energy propagation through the cured structure and its penetration into the longitudinal microchannel during the fabrication of the “Top Part”. To approximate realistic printing conditions, the energy penetration depth $D_{p}$ was set to 119.45, based on the “Working Curve” model determined values. To simplify the model configuration while maintaining computational feasibility, the layer thickness was set to 100 µm during the COMSOL simulation. The initial investigation focused on UV energy penetration along the YZ cross-section of the microchannel. As illustrated in Figure 4b, during the printing of the top first layer, UV energy continued to propagate into the longitudinal channel direction despite the completion of the initial layer’s curing. Although the overall penetration depth appeared relatively uniform, the spatial energy distribution within the microchannel exhibited pronounced irregularities and unpredictable patterns. This spatial variability poses considerable challenges to achieving micron-scale printing precision, highlighting the intrinsic limitations of the “Photopolymer Resin Exposure Model” in describing dynamic energy propagation behaviors. Notably, this irregular energy distribution was observed through COMSOL simulations for the first time in the context of DLP-printed microchannels. As the printing process advanced, the completion of the top second layer (Figure 4c) led to further attenuation of the UV energy, though partial energy penetration into the microchannel remained discernible. Compared to the top first layer, the energy intensity was notably reduced; however, simulation results indicated that a substantial portion of UV energy still traversed the top layer structure and was subsequently absorbed by the resin within the microchannel. Similarly, during the fabrication of the top third layer (Figure 4d) and top fourth layer (Figure 4e), partial UV energy penetration into the microchannel persisted despite the progressive decline in energy intensity with each additional layer. By the time the top fifth layer was printed (Figure 4f), simulation results revealed that although UV energy penetration still occurred, its intensity was significantly diminished after traversing the previously cured layers. At this stage, minimal energy reached the microchannel interior, rendering its contribution to resin curing negligible. This result underscores the existence of a critical thickness in the printing process. Once the cumulative thickness of the printed structure exceeds this threshold, UV energy penetration becomes inconsequential due to pronounced energy attenuation, effectively mitigating its influence on resin curing within the microchannel.

**Figure 5:**
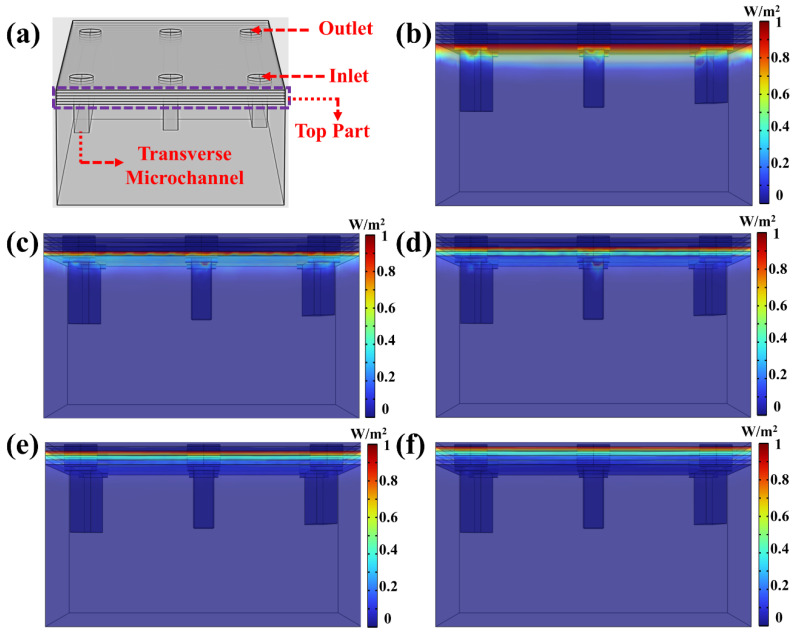
(**a**) Three-dimensional simulation of UV energy penetration in YZ cross-section of the linear microfluidic chip, (**b**–**d**) different printed top layer UV energy penetration: (**b**) top first layer, (**c**) top second layer, (**d**) top third layer, (**e**) top fourth layer, (**f**) top fifth layer.

Most existing studies on microchannel over-curing have focused primarily on the longitudinal direction, with limited attention given to energy penetration in the transverse direction. However, microchannels in DLP-printed microfluidic chips inherently form three-dimensional structures, rendering a unidirectional analysis insufficient for capturing the complete dynamics of UV energy propagation. Evaluating energy penetration solely along the longitudinal axis provides a partial and scientifically inadequate representation of the curing process. To address this limitation, this study extends the simulation to the transverse cross-section of the microchannel, as depicted in Figure 5a. This approach facilitates a more comprehensive investigation of UV energy penetration and its cumulative effects during printing. Figure 5b illustrates the UV energy-penetration behavior in the transverse direction during the printing of the top first layer. Similar to the longitudinal direction, a significant amount of UV energy penetrates into the microchannel. However, its spatial distribution exhibits pronounced irregularity and randomness, characterized by high unpredictability. This stochastic energy distribution complicates efforts to achieve consistent and precise microchannel fabrication.

As the printing process advances, with successive printing of the top second, top third, and top fourth layers, the cumulative printed thickness progressively increases. As shown in Figure 5c–e, the intensity of UV energy penetrating the microchannel continuously decreases as additional layers are printed. By the time the top fifth layer is completed, the majority of UV energy has been absorbed or attenuated while passing through previously cured layers (Figure 5f). At this stage, minimal energy reaches the microchannel interior, rendering its impact on resin curing negligible. The simulation results reveal that UV energy penetration trends in both the transverse and longitudinal microchannel directions exhibit similar characteristics, including spatial randomness and irregular energy distribution. This unpredictable energy distribution is challenging to model using conventional mathematical frameworks but plays a critical role in determining microchannel printing accuracy. These findings underscore the need for advanced modelling approaches that integrate three-dimensional energy propagation dynamics to enhance the precision of microchannel fabrication.

### 3.3. Three-Dimensionl Modelling of Photopolymerization in DLP 3D Printing

During the DLP 3D printing process of microfluidic chips, continuous UV energy penetration is the primary cause of microchannel over-curing. However, UV energy penetration itself is only a triggering process; the absorbed energy initiates a series of resin phase transitions, transforming the resin from a liquid to a gel-like intermediate state and ultimately into a fully cured solid. This sequential curing process leads to microchannel over-curing. Therefore, after determining the UV energy penetration pattern and distribution during the printing process, it becomes essential to simulate how energy penetration affects resin curing within the microchannel on a layer-by-layer basis.

**Figure 6:**
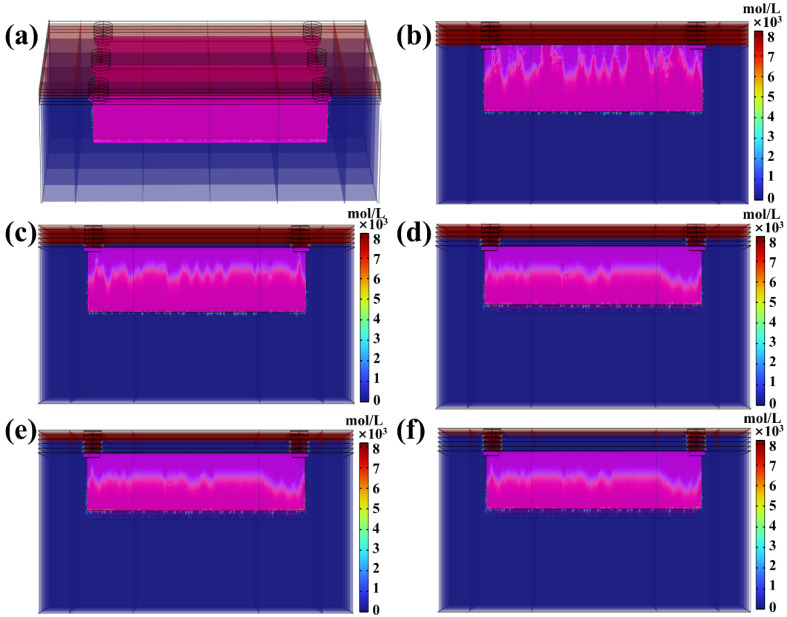
(**a**) Three-dimensional photopolymerization analysis in YZ cross-section of the linear microfluidic chip, (**b**–**d**) photopolymerization-based different printed top layers: (**b**) top first layer, (**c**) top second layer, (**d**) top third layer, (**e**) top fourth layer, (**f**) top fifth layer.

The “Domain ODEs and DAEs” module in COMSOL was employed to simulate the resin curing behavior during microchannel printing under UV energy penetration. In this simulation, the degree of resin curing is quantitatively represented by the monomer concentration within the resin. Initially, when no UV energy is absorbed, the resin remains in a liquid state with the monomer concentration at its maximum. As UV energy is progressively absorbed, the resin transitions from a liquid to a gel-like state and ultimately solidifies, at which point the monomer concentration reduces to zero. This method provides a clear and quantitative characterization of the dynamic evolution of resin states during the curing process. Upon complete curing, the monomer concentration is set to zero. Tracking this concentration provides a direct visualization of the curing process. As shown in Figure 6a, in the longitudinal channel direction of the annular microfluidic chip, the “Substrate Part” and “Microchannel Part” have already been printed, appearing in blue, indicating a monomer concentration of zero and thus fully cured regions. Conversely, the microchannel interior remains red, indicating liquid resin with the maximum monomer concentration. As the “Top Part” structure printing proceeds, the resin within the microchannel begins to cure. Upon completing the top first-layer printing (Figure 6b), some resin inside the microchannel absorbs UV energy and transitions to a gel-like state. However, rather than forming a uniform cured layer, the curing process exhibits uneven energy absorption, leading to scattered, independent gel-like resin clusters. With the printing of the top second layer, these previously formed clusters merge as the remaining liquid resin continues to cure, creating a continuous over-cured structure and increasing its overall thickness. Upon printing the top second layer (Figure 6b) and top third layer (Figure 6d), the over-cured layer’s thickness continues to increase, albeit at a slower rate. By the time the top fourth layer is printed (Figure 6e), the thickening trend slows considerably. Finally, after completing the top fifth layer, the residual UV energy is nearly entirely attenuated by passing through the previously printed layers (Figure 6f). As a result, minimal UV energy reaches the microchannel, causing the over-cured thickness to stabilize without further expansion.

**Figure 7:**
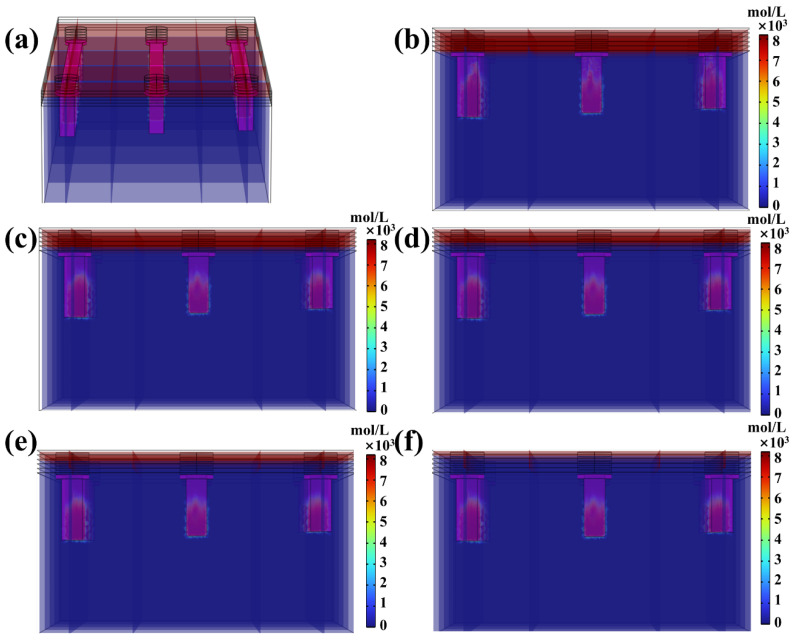
(**a**) Three-dimensional photopolymerization analysis in XZ cross-section of the linear microfluidic chip, (**b**–**d**) photopolymerization-based different printed top layers: (**b**) top first layer, (**c**) top second layer, (**d**) top third layer, (**e**) top fourth layer, (**f**) top fifth layer.

UV energy penetration causing microchannel over-curing in the transverse channel direction also warrants detailed investigation. As depicted in Figure 7a, the resin inside the transverse microchannel appears red, indicating that the resin remains in a liquid state with no curing initiated. Upon completing the top first-layer printing (Figure 7b), UV energy penetrates the microchannel, triggering resin curing. The resin gradually transitions from a liquid state (red) to a gel-like state (approaching blue) as it absorbs the penetrated UV energy. Notably, the curing process exhibits significant spatial irregularity, with three identically sized microchannels displaying distinctly different curing distributions. Further inspection reveals that, similar to the longitudinal channel direction, the cured resin structures inside the transverse microchannel also form scattered, gel-like clusters. This observation underscores the inherent randomness and unpredictability associated with UV energy penetration-induced over-curing, posing substantial challenges to achieving high-precision microchannel printing. Figure 7c illustrates the curing state after printing the top second layer. Compared to Figure 7b, the previously observed clustered gel-like structures have merged into a more continuous and integrated cured layer, accompanied by an increase in over-curing thickness. As the printing process advances, the printing of the top third layer (Figure 7d) and top fourth layer (Figure 7e) further increases the over-curing thickness, though the thickening rate noticeably declines. Finally, after printing the top fifth layer (Figure 7f), the over-curing thickness within the microchannel stabilizes, and no further changes are observed. This behavior aligns with the UV energy penetration trend previously noted in the longitudinal channel direction.

### 3.4. Three-Dimensional Modelling of Particle Tracing in DLP 3D Printing

**Figure 8:**
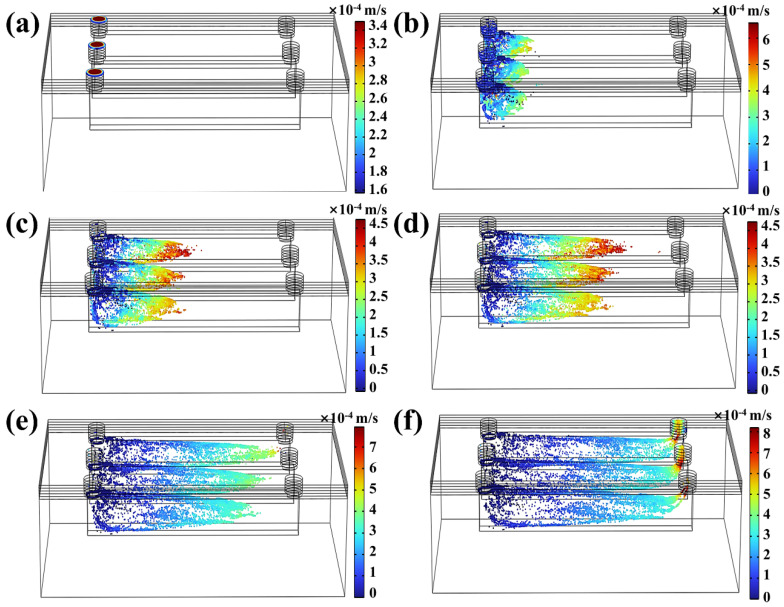
Dynamic 3D particle tracing in the linear microfluidic chip. (**a**) Initial moment of structure–film separation, (**b**–**d**) particle dynamics at different stages of film separation: (**b**) 0.2 s after separation, (**c**) 0.4 s after separation, (**d**) 0.6 s after separation, (**e**) 0.8 s after separation, (**f**) 1.0 s after separation.

While UV energy penetration has been widely recognized as the primary cause of over-curing during DLP microchannel printing, resin flow dynamics also play a critical role in shaping the final over-cured structures of the microchannel. However, the “Photopolymer Resin Exposure Model” primarily considers UV energy propagation, neglecting the dynamic movement of resin within the microchannel during printing. In reality, each printing layer involves a peeling process between the release film and the cured structure, generating a pressure differential between the microchannel’s inlet and outlet. This pressure differential induces directional resin flow within the microchannel, significantly influencing the curing profile. As illustrated in Figure 8, to capture the dynamic behavior of resin flow during the peeling process, the “Particle Tracing for Fluid Flow” module in COMSOL was utilized for simulation analysis. In this simulation, the separation time between the cured structure and the release film was set to 1 s to accurately reflect the resin flow characteristics and its temporal evolution during the printing process. In Figure 8a, immediately after the inlet separates from the release film, a pressure difference develops, causing an initial velocity shift near the inlet. As the peeling process progresses (Figure 8b), directional resin flow becomes evident along the longitudinal channel direction. Figure 8c–e further depict the lateral movement of resin within the microchannel driven by the continuous peeling action. The peeling process concludes when the release film entirely separates from the outlet, as shown in Figure 8f, allowing a portion of the resin to exit through the outlet and complete a directional flow cycle within the microchannel. Unlike UV energy penetration, which diminishes as more layers are printed, the peeling-induced flow persists throughout the entire printing process due to the DLP system’s “Layer-by-Layer” printing nature. This periodic resin movement causes partially gelled resin to shift directionally within the microchannel, further amplifying the randomness and unpredictability of microchannel over-curing, complicating efforts to achieve precise and uniform printing results.

**Figure 9:**
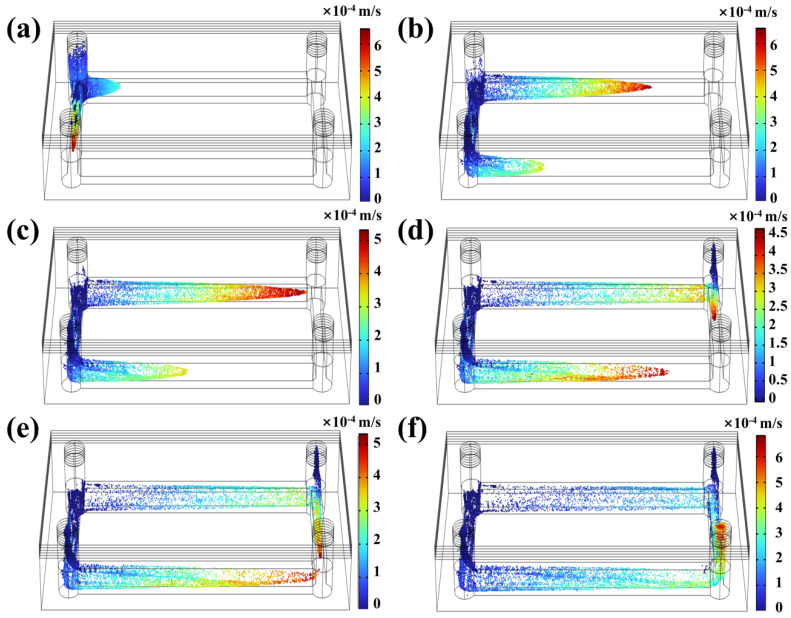
Dynamic 3D particle tracing in the annular microfluidic chip. (**a**) Initial moment of structure–film separation, (**b**–**d**) particle dynamics at different stages of film separation: (**b**) 0.2 s after separation, (**c**) 0.4 s after separation, (**d**) 0.6 s after separation, (**e**) 0.8 s after separation, (**f**) 1.0 s after separation.

The separation process between the release film and the cured structure significantly affects the directional resin flow within the microchannel, with structural design playing a crucial role. To explore this effect, Figure 9 illustrates resin flow patterns and characteristics during the separation of the annular microfluidic chip from the release film. Unlike the conventional linear microfluidic chip, the annular microfluidic chip features a ring-shaped microchannel comprising four symmetrically arranged channels, enabling a more comprehensive observation of multidirectional resin flow behavior. The annular microfluidic chip has two inlets and two outlets, producing distinct spatial flow patterns during separation. As shown in Figure 9a, the resin begins to flow in two directions when the release film first detaches from one of the inlets. Upon further separation from the second inlet (Figure 9b), three microchannels exhibit evident resin movement. As the peeling process continues, resin progressively flows toward the outlets, as depicted in Figure 9c,d. When the release film separates from the first outlet (Figure 9e), directional resin flow emerges in the fourth microchannel. Finally, once the release film fully detaches from the cured structure, a portion of the resin exits through the last outlet (Figure 9f). Combining the effects of UV energy penetration observed in previous simulations, it becomes apparent that directional resin flow significantly impacts the curing process. As partially gelled resin migrates predictably along fixed paths, the periodic peeling action caused by DLP’s “Layer-by-Layer” printing process induces recurrent directional resin movement toward specific microchannel regions. This periodic flow accumulates resin along predefined trajectories, altering the final cured structure. Therefore, incorporating previous simulation findings from “Photopolymerization” and “UV Energy Penetration”, it can be inferred that the final over-curing profile is unlikely to be an irregular flat cross-section as initially assumed. Instead, the resin-flow dynamics induce an inclined cross-sectional profile with distinct spatial patterns, driven by the continuous interaction between UV energy penetration and resin movement during the printing process.

### 3.5. Microfluidic Chips Print Based on DLP

By integrating and analyzing COMSOL simulations based on “General from PDE”, “Domain ODEs and DAEs”, and “Particle Tracing for Fluid Flow” modules, it was determined that resin flow within the microchannel and UV energy penetration into the cured structure are concurrent dynamic processes during DLP-based microfluidic chip printing. Consequently, the final over-cured structure’s thickness is influenced not only by the progressive increase in the “Top Part” thickness but also by the directional resin flow induced by the repeated detachment between the cured structure and the release film. Based on this dynamic interaction, we hypothesize that the combined effects of UV energy penetration and resin flow significantly affect the formation of over-cured structures within the microchannel. As resin undergoes repeated directional flow within the microchannel, partially received UV penetration resin accumulates regularly at different locations, eventually forming a patterned, non-uniform cross-sectional profile.

**Figure 10:**
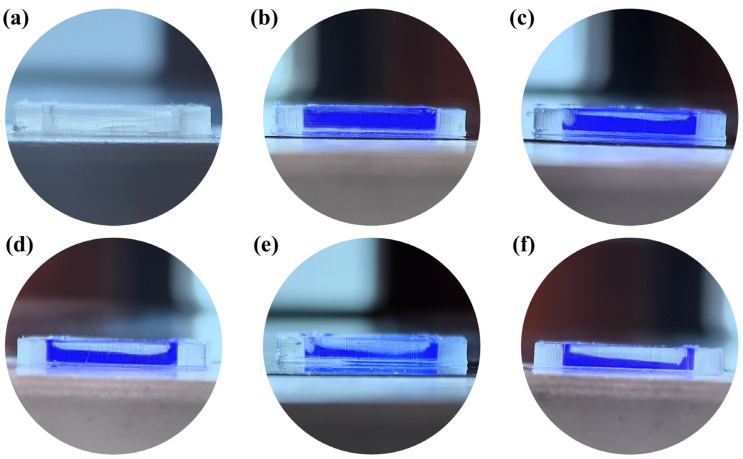
(**a**) Printed linear microfluidic chip based on DLP, (**b**–**d**) the microchannel shape with different top part-structure thicknesses: (**b**) 100 µm, (**c**) 200 µm, (**d**) 300 µm, (**e**) 400 µm, (**f**) 500 µm.

To experimentally validate this hypothesis, linear microfluidic chips and annular microfluidic chips were printed using a custom-built DLP 3D printer. As shown in Figure 10a, the linear microfluidic chip was fabricated, featuring a transparent structure that facilitates direct observation of internal microchannels. To visualize the printed structures more clearly, a synthesized blue solution was injected into the microchannels, where the blue regions indicate liquid-filled microchannels, while the white transparent areas correspond to over-cured resin structures. As the thickness of the “Top Part” structure increased, the over-cured structure within the microchannel gradually exhibited an inclined profile. To quantify this behavior, the endpoint of the over-cured layer was defined as the true over-cured thickness of the microchannel, while the maximum inclination of the over-cured structure was defined as the over-cured inclination angle. As shown in Figure 10b, when the “Top Part” thickness was set to 100 µm, the microchannel exhibited a relatively thin over-cured layer with a flat cross-section, corresponding to an over-cured thickness of 83 µm and an inclination angle of 0°. However, when the “Top Part” thickness increased to 200 µm (Figure 10c) and 300 µm (Figure 10d), the over-cured layer thickened to 218 µm and 483 µm, respectively, and the cured surface developed an inclined profile, with inclination angles of 8.4° and 10.3°, respectively. Further increases in the “Top Part” thickness to 400 µm (Figure 10e) and 500 µm (Figure 10f) resulted in significant growth of the over-cured layer thickness to 691 µm and 804 µm, respectively, accompanied by pronounced inclined cross-sections. The corresponding inclination angles increased further to 13.9° and 16.1°. Overall, both the over-cured thickness and inclination angle exhibited a clear upward trend with increasing “Top Part” thickness.

**Figure 11:**
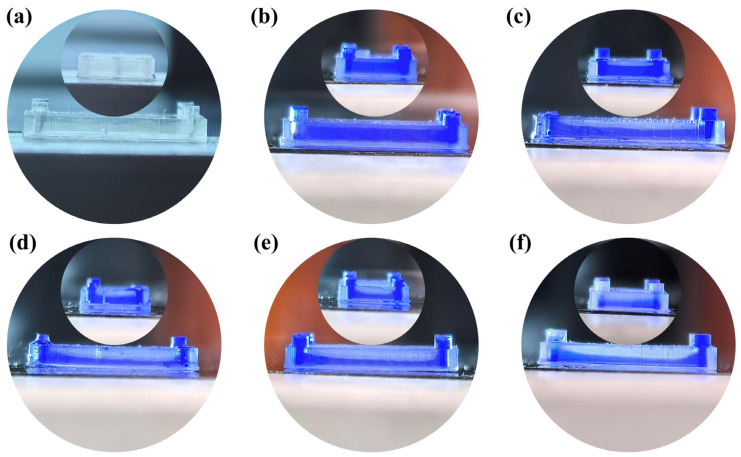
(**a**) Printed annular microfluidic chip based on DLP. (**b**–**d**) The microchannel shape with different top part structure thicknesses: (**b**) 100 µm, (**c**) 200 µm, (**d**) 300 µm, (**e**) 400 µm, (**f**) 500 µm.

To further verify the hypothesis, an annular microfluidic chip was printed using identical fabrication parameters. Due to its unique ring-shaped design, over-curing was evaluated along both longitudinal and transverse microchannel directions, as shown in Figure 11a. Figure 11b illustrates the microchannel cross-section when the “Top Part” thickness was 100 µm, showing moderate over-curing with relatively flat surfaces in both directions. At this stage, the over-cured thickness in the longitudinal and transverse directions was 91 µm and 64 µm, respectively. As the “Top Part” thickness increased to 200 µm (Figure 11c), 300 µm (Figure 11d), and 400 µm (Figure 11e), the over-cured thickness in the longitudinal direction increased to 243 µm, 647 µm, and 804 µm, with inclination angles rising from 5.9° to 10.8° and 11.3°. Meanwhile, in the transverse direction, the over-cured thickness grew to 169 µm, 264 µm, and 593 µm, with inclination angles increasing from 2.1° to 3.9° and 6.7°. Overall, the longitudinal direction consistently exhibited a more pronounced inclination angle compared to the transverse direction. When the “Top Part” thickness reached 500 µm (Figure 11f), significant inclination was observed in both directions. At this stage, the transverse over-cured thickness and inclination angle were 647 µm and 8.1°, respectively, while the longitudinal over-cured thickness and inclination angle reached 804 µm and 16.1°, indicating a steeper slope in the longitudinal direction. This discrepancy can be attributed to the shorter resin flow path in the transverse direction, which restricts fluid movement and reduces the extent of resin redistribution. Therefore, due to the longitudinal direction exhibiting a more distinct inclined profile because of longer resin flow paths and greater fluid displacement, when comparing the longitudinal direction over-cured structure, the over-cured structures in the transverse direction remained comparatively flatter. Similarly, by analyzing the evolution of over-cured structures in the printed linear and annular microfluidic chips, it was found that as the “Top Part” thickness increased, the over-cured layer thickened, and the microchannel profile gradually developed an inclined geometry. These findings are in excellent agreement with the predictions from the DLP-OCIM model, accurately validating that the formation of over-cured structures within microchannels results from the coupled effect of UV energy penetration and directional resin flow.

## 4. Discussion

In this study, the DLP-OCIM model was established using COMSOL to investigate the coupling mechanism between UV energy penetration and directional resin flow within microchannels during the “Top Part” structure printing process. The model revealed how these interactions jointly influence the formation of over-cured structures. To validate this hypothesis proposed by the DLP-OCIM model, linear and annular microfluidic chips with varying “Top Part” thicknesses were designed, printed, and analyzed. The experiment showed that with the “Top Part” thickness increased, the thickness of the over-cured structure was gradually thicker while its cross-sectional profile evolved from a flat structure to an increasingly sloped geometry (Figure 10b–e and Figure 11b–e). This simulation and verification experiment marks the first detailed investigation into microchannel over-curing formation, successfully filling a previous research gap that primarily focused on vertical dimensional accuracy while neglecting cross-sectional shape fidelity.

Given that identical DLP printing parameters, photopolymer resin, and device settings were applied to print both linear and annular microfluidic chips, according to the traditional “Photopolymer Resin Exposure Model”, the two chip designs should exhibit similar over-cured structures due to identical exposure energy, with uniform cross-sectional shapes based on UV energy distribution, such as flat or Gaussian profiles. However, as the “Top Part” thickness increased, the over-cured cross-sectional profiles in both chip types transitioned from flat to sloped geometries (Figure 10 and Figure 11). This divergence can be attributed to UV energy penetrating the cured layers during exposure (Figure 4 and Figure 5), followed by directional resin flow caused by the peeling process between the release film and the cured structure (Figure 8 and Figure 9). As the printed layers accumulated, partially gelled resin subjected to periodic resin flow continuously migrated within the microchannel, forming inclined over-cured structures in specific regions. Additionally, a comparison of over-curing patterns between linear and annular microfluidic chips revealed that even under identical UV exposure, distinct microchannel designs result in different over-curing profiles. The linear chip exhibited a more pronounced inclined over-cured structure than the annular chip due to differences in microchannel geometry. As indicated by the particle tracing simulation, the resin flow in the linear chip followed a straightforward path from a single inlet to an outlet (Figure 8). In contrast, resin flow in the annular chip involved directional changes and longer flow paths, reducing the amount of resin reaching the outlet and resulting in a gentler inclined cross-section (Figure 9). Moreover, directional differences in over-curing were also observed within the same chip. As shown in Figure 11d–f, the longitudinal microchannels of the annular chip exhibited steeper slopes than the transverse microchannels. This difference is explained by the shorter flow path of the transverse microchannels, which limits resin flow and diminishes directional movement. Consequently, the transverse channels exhibited flatter over-cured structures compared to the longitudinal channels.

Overall, the integrated results of the COMSOL simulations and microfluidic chip experimental print verify that microchannel over-curing is dominated by UV energy penetration as well as the resin flow dynamics of the microchannel during the DLP printing process. The extent of resin flow is significantly influenced by the microchannel’s geometric design, including parameters such as height, width, and shape. Therefore, future research will focus the synthesis of photopolymers with lower UV penetration depths to further minimize energy infiltration and limit over-curing effects. Furthermore, the various microchannel structures will be optimized and designed to control resin flow and control the flow path, to limit the generation of the inclined over-cured cross-section. Additionally, improving the surface roughness of the release film, or modifying the cured structure surface could further decrease pressure differentials during the peeling process, suppressing resin flow dynamics and enabling higher-precision printing of complex microchannel geometries. Alternatively, some novel printing process that could prevent UV penetration energy from entering the cured structure from the “Top Part” entirely may be developed, offering a direct solution to mitigate the over-curing effects.

## 5. Conclusions

This study developed the 3D DLP-OCIM model using COMSOL, integrating UV energy penetration, photopolymerization, and particle-tracking modules to simulate energy propagation, resin flow, and curing processes within microchannels during DLP-based microfluidic chip printing. This model especially focused on UV energy penetration induced by the layer-by-layer fabrication process of the “Top Part” structure while also capturing the flow dynamics of the resin caused by the detachment of the cured structure from the release film. Analyzing the simulation results, this is the first study proposing a coupling mechanism for the interaction between UV energy penetration into microchannel wells and directional resin flow during DLP printing, elucidating how they together influence the over-curing of structures. As printing progresses, the combined effect of energy penetration and resin flow gradually leads to the formation of over-cured structures with increasingly inclined cross-sectional profiles, significantly impacting microchannel printing precision. To validate this hypothesis, the linear and annular microfluidic chips with different “Top Part” thicknesses were designed and printed, and the over-cured structure of the microchannel was systematically analyzed. These experiments confirmed that as the “Top Part” thickness increased, the cross-sectional profile of the over-cured section gradually evolved from an initially flat structure to a pronounced inclined shape, with the inclination angle increasing proportionally to the “Top Part” thickness. This experiment was consistent with simulation predictions. This study is the first to reveal the dynamic coupling effect between UV energy penetration and the resin flow of microchannels through both simulation and experimental validation. By establishing a fully 3D DLP-OCIM model, the study highlights the critical role of UV energy propagation and resin flow dynamics in shaping over-cured structures during microchannel printing, offering valuable theoretical insights and practical design optimization strategies for printing precise microfluidic chips and other hollow microchannel devices based on DLP technology.

## Figures and Tables

**Table 1:** Parameter definitions of COMSOL simulation.

Parameter Definitions	Value	Data Source
Resin density, ρ	1100 [kg/$m^{2}$]	Technical data sheet
Dynamic viscosity of resin, μ	280 [cP]	Technical data sheet
Light penetration depth, $D_{p}$	125.45 × $10^{-6}$ [m]	Experiment
Polymerization lumped rate constant, ℙ	0.15 [$m^{2}/W$]	[42]
Particle density, $\rho_{p}$	2200 [kg$/m^{3}$]	-
Particle diameter, $d_{p}$	0.1 [nm]	-
Maximum light intensity, $I_{max}$	27.2 × $10^{-3}$ [$W/m^{2}$]	Experiment
Initial light intensity, $I_{0}$	0 [$W/m^{2}$]	-
Initial monomer concentration, ${\left[M\right]}_{0}$	8.2 [mol/L]	[42]
Inlet velocity, $u_{0}$	0.35 [mm/s]	[27]
Layer thickness	100 µm	

## Data Availability

Data will be made available on request.
